# Supplementary material for: Endoplasmic Stress Affects the Coinfection of Leishmania Amazonensis and the Phlebovirus (Bunyaviridae) Icoaraci
Source: Viruses. 2022 Sep 2;14(9):1948. doi: 10.3390/v14091948 (PMC9503334; doi:10.3390/v14091948)
Supplement: Supplementary file 1 [file viruses-14-01948-s001.zip › Supplementary Figures.pdf]

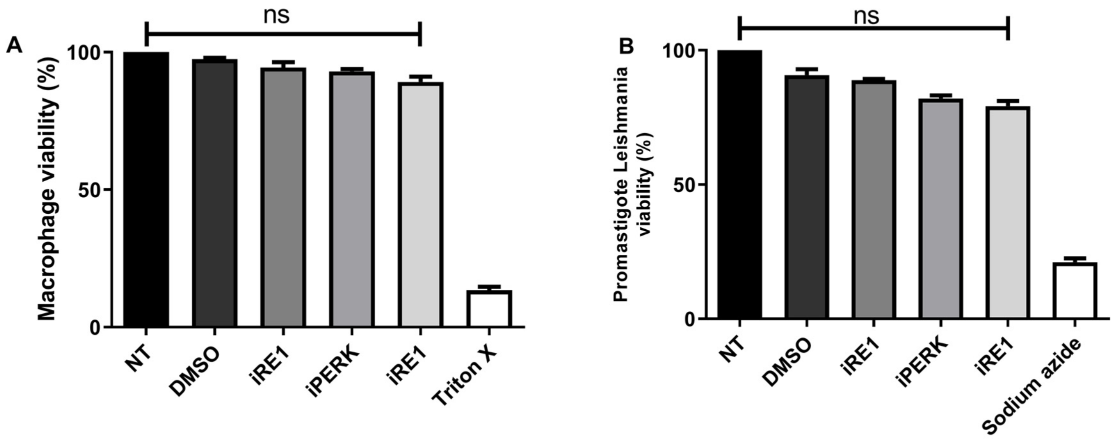

Figure S1. ER stress inhibitors do not affect Leishmania viability.

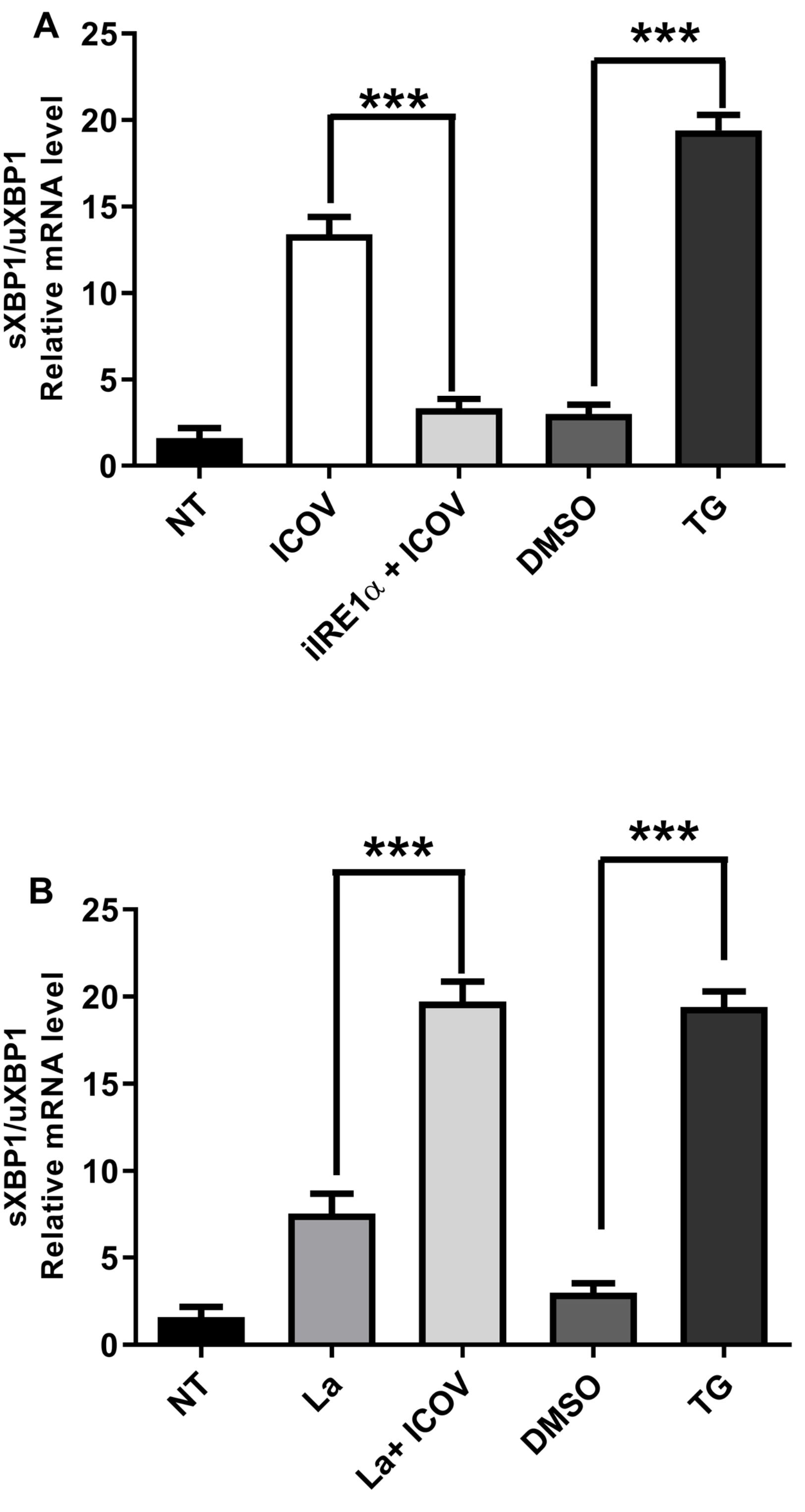

Figure S2. XBP1 splicing is maintained for 24 h post-coinfection.

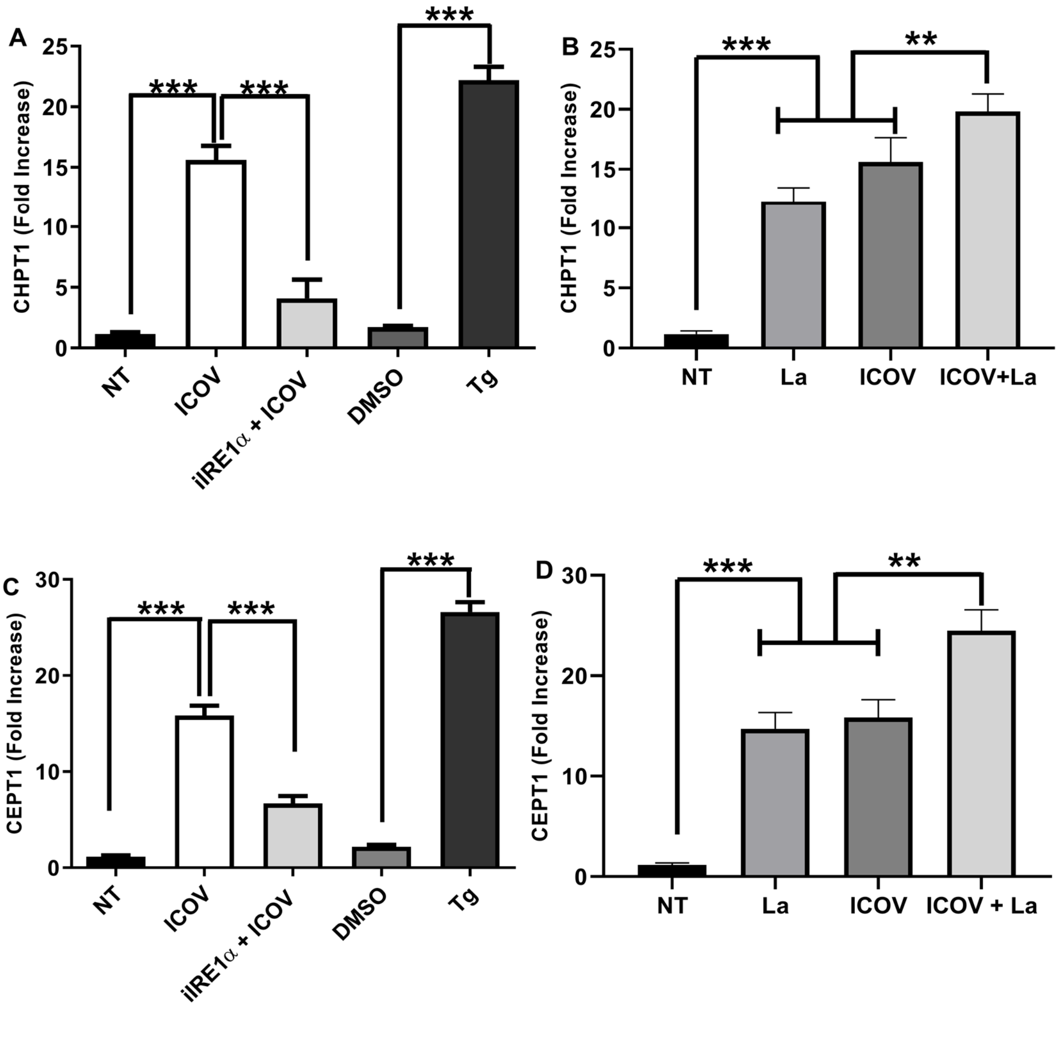

Figure S3. The upregulation of CHPT1 and CEPT1 is sustained for 24 h post-coinfection.

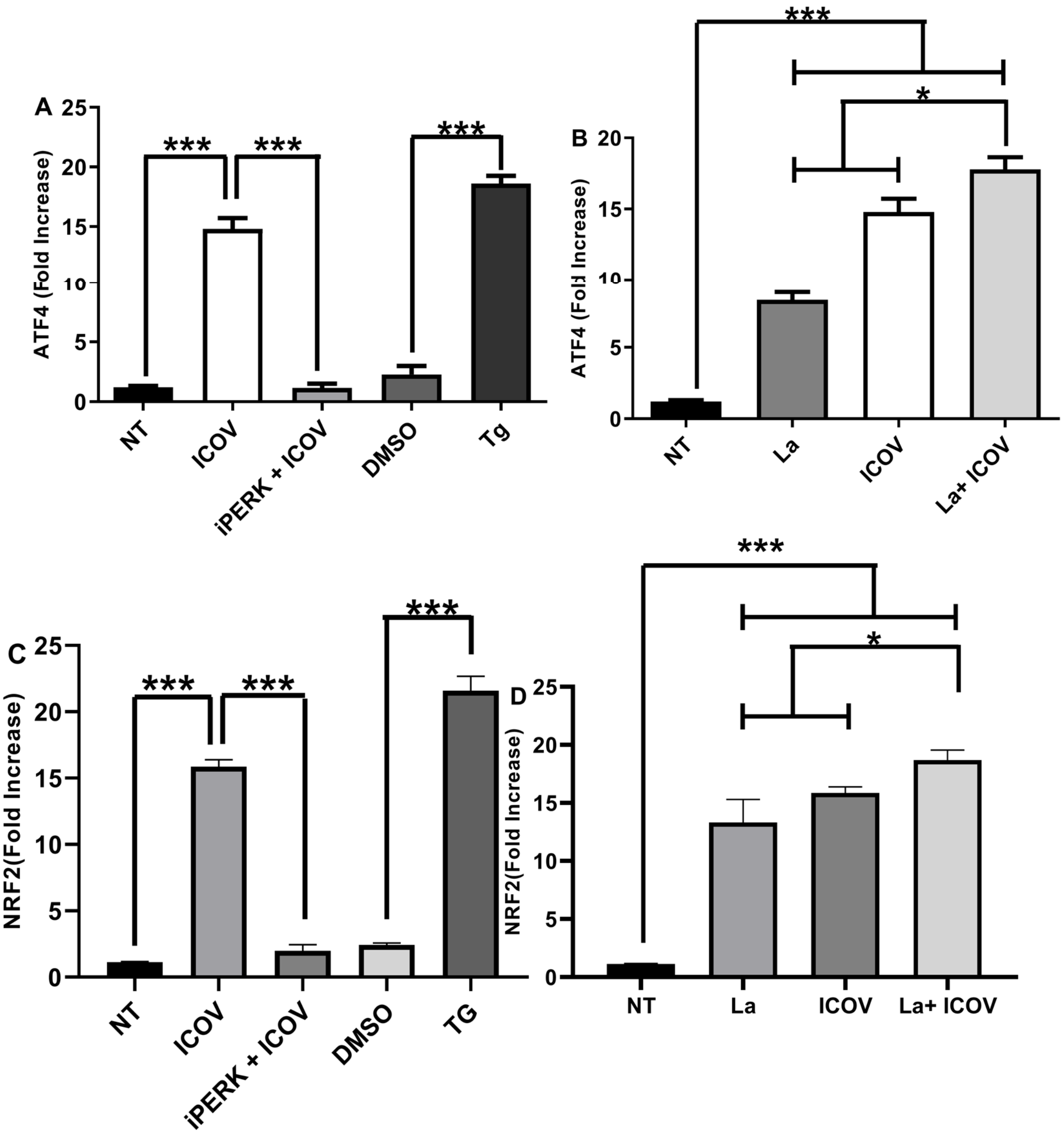

Figure S4. The expression of ATF4 and NRF2 is sustained for 24 h post-coinfection.
